# Brief mindfulness-based meditation enhances the speed of learning following positive prediction errors

**DOI:** 10.1177/17470218241228859

**Published:** 2024-02-14

**Authors:** Marius Golubickis, Lucy B G Tan, Parnian Jalalian, Johanna K Falbén, Neil C Macrae

**Affiliations:** 1 The School of Psychology, University of Aberdeen, Aberdeen, UK; 2 Clinical Psychology, School of Social and Health Sciences, James Cook University, Singapore; 3 Department of Psychology, University of Amsterdam, Amsterdam, The Netherlands

**Keywords:** Probabilistic learning, mindfulness-based meditation, prediction errors, computational modelling

## Abstract

Recent research has demonstrated that mindfulness-based meditation facilitates basic aspects of cognition, including memory and attention. Further developing this line of inquiry, here we considered the possibility that similar effects may extend to another core psychological process—instrumental learning. To explore this matter, in combination with a probabilistic selection task, computational modelling (i.e., reinforcement drift diffusion model analysis) was adopted to establish whether and how brief mindfulness-based meditation influences learning under conditions of uncertainty (i.e., choices based on the perceived likelihood of positive and negative outcomes). Three effects were observed. Compared with performance in the control condition (i.e., no meditation), mindfulness-based meditation (1) accelerated the rate of learning following positive prediction errors; (2) elicited a preference for the exploration (vs. exploitation) of choice selections; and (3) increased response caution. Collectively, these findings elucidate the pathways through which brief meditative experiences impact learning and decision-making, with implications for interventions designed to debias aspects of social-cognitive functioning using mindfulness-based meditation.

The benefits of an open mind are considerable. Receptive to the complexities of daily life, open minds display a willingness to consider competing viewpoints, entertain alternative perspectives, and process information in an even-handed manner. In contrast, when closed, minds tend to be dogmatic, cognitively inflexible, and over-reliant on pre-existing opinions, assumptions, and beliefs (Eagly et al., 1999; Nickerson, 1998; Price et al., 2015). Despite these downsides, it should be noted that closed minds can nevertheless offer consistency, cognitive economy, and clarity when multiple choice-related options are available. Practically speaking, these states of mind exert distinct effects on decisional processing (Smillie, 2017). Whereas open-minded cognition is amenable to the potential value of previously untried options/choices, closed-minded thinking is dominated by tried-and-tested decisions that have been successful in the past. As such, the primary advantage of an open mind is that it acknowledges the existence of rival possibilities, thereby optimising decision-making in various task environments. So, relatively speaking, when are minds more open than closed?

Reflecting a core dimension of personality (McCrae, 1993), people high (vs. low) in trait “openness to experience” exhibit many of the upsides of flexible cognition (DeYoung, 2013), in that they tend to be inquisitive, creative, and eager to explore the world and its myriad possibilities (Kaufman et al., 2016; McCrae & Costa, 1997; Silvia et al., 2008). For example, outside the laboratory, these individuals are more likely to visit galleries and museums, possess impressive literary collections, and have spent longer in formal education than their cognitively rigid peers (e.g., Carney et al., 2008; Chamorro-Premuzic et al., 2011; Gosling et al., 2002; Trapp & Ziegler, 2019; Van Eijck & De Graaf, 2004). In many (but not all) task settings, openness to experience is associated with curiosity, information seeking, and the development of personal knowledge (Smillie, 2017).

Pertinent to the current investigation, open-minded cognition arises from factors other than the possession of specific personality characteristics, with situational forces and temporary psychological states also playing a significant contributory role (Jach et al., 2022; Sutton & Barto, 1998). Notably, strategies that promote mindfulness have been shown to cultivate cognitive flexibility and an openness to new experiences. Practised for centuries, mindfulness is a major component of contemporary functional contextual therapies, such as the acceptance and commitment therapy (ACT; Hayes et al., 2012). Originating in eastern Buddhist philosophies (Hayes, 2002), this practice can take many forms, including guided meditation, mindful breathing, mindful movement, mindful eating, and progressive body scan meditation. Operationally, these techniques moderate the salience of internal/external experiences (Luoma et al., 2007) and have acknowledged effectiveness in reducing a range of psychological symptoms (e.g., anxiety, depression, and stress; Carmody & Baer, 2008; Chiesa & Serretti, 2009; Vøllestad et al., 2012). In addition, through the non-judgmental evaluation of present-moment thinking (Baer et al., 2006; Bishop et al., 2004; Brown & Ryan, 2003; Kabat-Zinn, 2003), mindfulness-based practices also impact core psychological processes. For example, even among novice meditators, as little as 5–10 min of experimentally induced mindfulness is sufficient to influence emotional appraisal, selective attention, action control, social perception, and self-construal (e.g., Erisman & Roemer, 2010; Farb et al., 2007; Golubickis et al., 2016, 2022; Jha et al., 2007; Papies et al., 2012, 2015; Tan et al., 2014).

By decreasing judgmental impulsivity and underscoring the value of novel experiences (Baer et al., 2006; Dixon et al., 2019; Hölzel et al., 2011), we suspect that mindfulness meditation may facilitate another fundamental psychological process—instrumental learning.^1^ In settings in which competing options are readily available, decision makers are confronted with a commonplace dilemma. Is it better to make choices that have been rewarding in the past (i.e., exploitation) or instead choose novel selections of uncertain value (i.e., exploration)? For example, when deciding on a Thai restaurant for date night, is it better to visit establishments that have been delightful in the past or take a chance on an unknown (i.e., riskier) eatery that could be wonderful or disappointing (Cohen et al., 2007)? Although optimal learning (i.e., which are the tastiest Thai restaurants in one’s neighbourhood?) calls for a balance between these competing strategies, at least in the long run, exploration tends to facilitate the acquisition of knowledge (Sutton & Barto, 1998).

Together with a range of other factors, openness to new experience exerts influence on this basic decision-making dilemma. Whereas individuals high in openness are generally receptive to novel experiences and ideas (even when faced with uncertainty), which inclines them towards exploration; those lower in openness prioritise the comfort of familiar choices, thereby gravitating towards exploitation (i.e., recently rewarded outcomes dominate decision-making). If, therefore, mindfulness attunes people to the potential value of competing alternatives, an interesting possibility arises. Mindfulness may trigger a preference for exploration (vs. exploitation) when learning takes place under conditions of uncertainty (Sternberg, 2002). In addition, given that mindfulness has been demonstrated to promote response caution (i.e., careful decision-making), learning in challenging task environments may be accompanied by an increase in the evidential requirements of choice selection (Golubickis et al., 2023; van Vugt & Jha, 2011; van Vugt & van den Hurk, 2017). That is, when mindful, additional evidence may be required before a response is selected.

Given these observations, our objectives in the current investigation were twofold. First, the primary objective was to explore the effects of brief mindfulness-based meditation (vs. no meditation) on instrumental learning. Specifically, we sought to establish whether the process of adjusting one’s behaviour based on prior choice-related outcomes (i.e., learning) changes following guided meditative practice. To investigate this issue, a probabilistic selection task (PST) was adopted in which participants’ reinforcement learning (RL) abilities were probed under conditions of uncertainty (Frank et al., 2004, 2007). The PST has been widely (and successfully) used to examine how people learn from positive versus negative choice-related feedback (i.e., reinforcement). Following a brief period of mindfulness-based meditation (vs. no meditation), participants were presented with three different stimulus pairs (i.e., AB, CD, EF; see Figure 1) comprising Japanese Hiragana characters. Their task was to figure out, based on repeated choice selections, which symbol in each pairing was most likely to be correct (Frank et al., 2004). Crucially, the feedback provided after each selection was probabilistic and varied across the stimulus pairs (i.e., AB = 80%–20%, CD = 70%–30%, EF = 60%–40%). For example, on AB trials, choosing symbol A led to positive feedback (i.e., correct) on 80% of trials, whereas selecting symbol B led to positive reinforcement on only 20% of the trials. Thus, over numerous choices, participants learned which item in each stimulus pair was more likely to be correct (e.g., A, C, E rather than B, D, F). We hypothesised that a brief period of mindfulness-based meditation would enhance the rate of learning during the PST.

**Figure 1.**
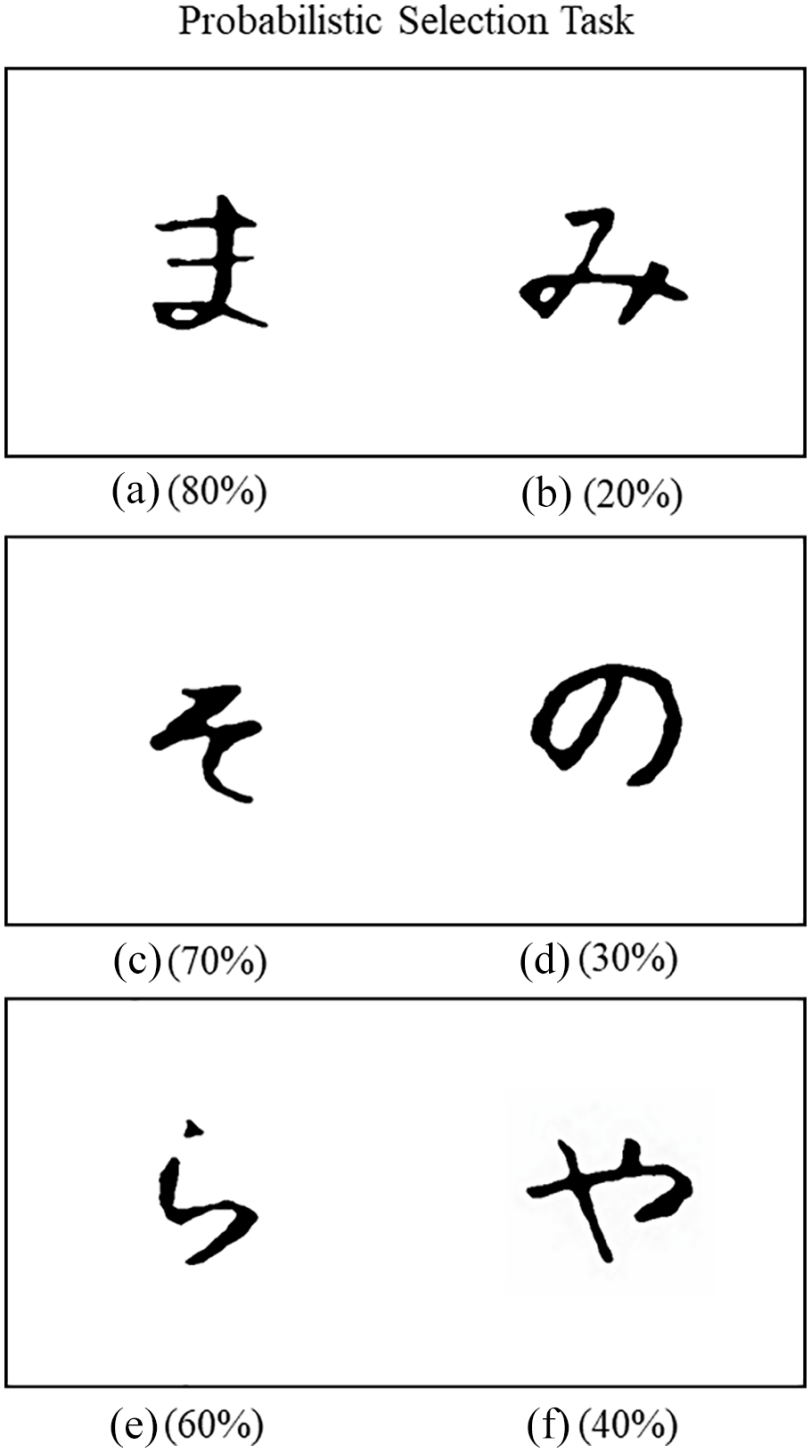
Example of the stimulus pairs (i.e., Japanese Hiragana characters) and the probabilities of correct responses during the probabilistic selection task.

Our second objective was to discern the underlying mechanisms responsible for any observed effects of brief mindfulness meditation on learning. To identify these processes, computational modelling was undertaken on the data (Golubickis & Macrae, 2022). Despite a substantial literature demonstrating the benefits of mindfulness on cognition and behaviour, quite how these effects arise remains less certain (but see Hölzel et al., 2011; Lutz et al., 2008; Papies et al., 2015; Shapiro et al., 2006; Tang et al., 2007; Teper & Inzlicht, 2013). In this respect, computational approaches are valuable as they provide a mechanistic account of the cognitive operations through which meditative experiences modulate decisional processing (Golubickis et al., 2023; van Vugt et al., 2019; van Vugt & Jha, 2011; van Vugt & van den Hurk, 2017). Accordingly, based on recent analytical developments, a reinforcement learning drift diffusion model (RL-DDM) analysis was adopted (Fontanesi et al., 2019; Pedersen et al., 2017; Pedersen & Frank, 2020).

Integrating sequential sampling and RL models, the RL-DDM identifies the processes that underpin learning and how these are fine-tuned as learning progresses (Miletić et al., 2020; Pedersen & Frank, 2020; Ratcliff et al., 2016). While RL models are useful at predicting changes in the proportion of choice probabilities over the course of learning, they do not account for differences in response latencies, a fundamental dimension of learning (e.g., as learning takes place, decision times decrease). In this respect, sequential-sampling models (e.g., drift diffusion model [DDM]; Ratcliff et al., 2016; P. L. Smith & Ratcliff, 2004) are informative as they predict decision-making by elucidating how response selection and latencies collectively arise from a common set of latent cognitive processes (e.g., rate of evidence accumulation, response caution). In so doing, the RL-DDM is an extension of classic RL models that offers a more precise understanding of the processes through which learning unfolds over time (Fontanesi et al., 2019; Miletić et al., 2020; Pedersen et al., 2017; Pedersen & Frank, 2020).

## Method

### Participants and design

A total of 60 participants (42 females, 17 males, 1 other; *M*_age_ = 23.00, *SD* = 2.96), with normal or corrected-to-normal visual acuity, took part in the research. Of the 60 participants, 30 (14 in the control condition) reported having minimal meditative experience (e.g., used an app once or twice). In terms of educational background, 36 held bachelor’s degrees, 12 master’s degrees, 2 PhDs, and 10 reported having a high-school education.^2^ Five participants (3 females, 2 males) failed to reach learning criteria (Frank et al., 2007), and thus were excluded from the analyses. Data collection was conducted online using Prolific Academic (www.prolific.co), with each participant receiving compensation at the rate of £8.00 (~US$10) per hour. Informed consent was obtained from participants prior to the commencement of the experiment and the protocol was reviewed and approved by the Ethics Committee at the School of Psychology, University of Aberdeen. The experiment had a single factor (Meditation: mindfulness or control) between-participants design. Based on prior research (Golubickis et al., 2023; Golubickis & Macrae, 2022), to detect a significant effect of meditation, a sample of 55 participants with a minimum of 60 learning trials afforded 86% power for a medium effect size (i.e., *d* = .50; PANGEA, v .0.2). This is a conservative estimation of the sample size as our analytic techniques (i.e., generalised estimating equations and RL-DDM) use hierarchical modelling. A key benefit of these approaches is that parameters are estimated reliably with a small number of experimental trials (Lerche et al., 2017; Wiecki et al., 2013). In addition, as models are fitted at the group-level rather than individually, larger participant numbers substantially improve parameter estimation.

### Stimulus materials and procedure

The experiment was performed online via Inquisit Web. Once participants accessed the experiment through the web link, they were randomly assigned to either the mindfulness or control condition. In the mindfulness condition, using headphones, participants listened to a 5-min audio-recording of a mindful breathing exercise based on foundational mindfulness-based stress reduction programmes. During this exercise, attention was focused on the sensation of breathing, with awareness directed in a non-reactive and non-elaborate manner to the present moment (Lymeus et al., 2018; Malinowski, 2013; Zanesco et al., 2018). Participants were asked to listen attentively to the recording and to avoid distractions (Golubickis et al., 2016, 2023; Tan et al., 2014; Tan & Martin, 2013, 2015). If, however, these arose they were requested to perceive the episodes as fleeting experiences and to return attention to their breathing each time a distracting thought, emotion, or memory occurred (H. Smith & Novak, 2003). A bell chimed after 5 min to signal the end of the activity. Prior research has established the effectiveness of this brief intervention in increasing levels of mindful-attention and awareness (Tan et al., 2014). In the control condition, participants performed a 5-min Chinese puzzle task (i.e., Tangram) in which they constructed shapes using polygons (Golubickis et al., 2023).

Next, all participants performed the learning phase of a PST (Frank et al., 2004, 2007). In this task, they were required to learn which symbol was more likely to be correct across three different symbol pairs (denoted as AB, CD, and EF, see Figure 1). After each selection, feedback was provided which showed whether the response was correct or incorrect. The probabilities indicating which symbol was more likely to be correct followed the standard version of the PST (Frank et al., 2004, 2007). Specifically, for the AB pair, A was 80% likely to be correct (20% for B), for the CD pair, C was 70% likely to be correct (30% for D), and finally, for the EF pair, E was 60% likely to be correct (40% for F). Over numerous choice selections, participants learned which item in each pairing was more likely to be correct (i.e., A, C, E rather than B, D, F) based on the feedback provided. The learning task finished when participants reached sufficient levels of accuracy for each pairing (i.e., AB, 60% or above; CD, 55% or above; EF, 50% or above; Frank et al., 2004, 2007).

Each trial began with the presentation of a pair of symbols that remained on the screen until the participant made a response. After the participant selected one of the symbols, both textual (i.e., the word “correct” in green or “incorrect” in red) and auditory (i.e., a high-pitched beep for a correct response or a low-pitched beep for an incorrect response) feedback were provided for 1,000 ms, followed by a blank screen for 500 ms, after which the next trial commenced. Participants had to select a symbol by pressing the appropriate button on the keyboard (i.e., “A” for the symbol on the left side of the screen, “L” for the symbol on the right side of the screen). The symbols in each pair were equally likely to be presented on the left or right side of the screen and stimulus presentation was randomised. Following previous research, no practice or familiarisation period was provided before the PST to prevent the possibility that prior understanding of the task would influence learning (Frank et al., 2004). Participants completed blocks of 60 trials in which each of the three stimulus pairs appeared randomly, equally often, until accuracy reached a satisfactory level. The maximum number of learning blocks was set to six (i.e., 360 trials in total) if the participant did not reach satisfactory levels of accuracy earlier in the task (Frank et al., 2007). If the participant’s learning performance was not sufficient after six blocks of trials, they were excluded from the analyses. On completion of the experiment, participants were debriefed and thanked.

### Computational analysis

The data from the PST were submitted to an RL-DDM analysis (Fontanesi et al., 2019; Pedersen et al., 2017; Pedersen & Frank, 2020).^3^ The RL-DDM estimates latent parameters through the simultaneous hierarchical Bayesian modelling of response time (RT) and choice data. A scaling parameter (i.e., drift rate, *v*_scaling_) measures sensitivity to choice-related feedback by taking both the expected outcome and speed of evidence accumulation into account, such that higher values indicate confident learning, whereas lower values imply uncertainty regarding the anticipated outcome. Drift rate scaling is equivalent to the inverse temperature parameter in classic instrumental learning models and indicates the extent to which exploration or exploitation is favoured during decision-making (Cohen et al., 2007; Daw et al., 2006; Pedersen et al., 2017; Sutton & Barto, 1998). Sensitive to the task context (Behrens et al., 2007), a learning rate parameter (*η*)—ranging from zero to one—quantifies how quickly learning takes place, with larger values indicating the utilisation of current feedback (i.e., fast learning), and smaller values reflecting reduced updating from recently experienced outcomes (i.e., slow learning). In this regard, either a single learning rate (*η*) that captures all learning, or separate learning rates for negative and positive prediction errors (*η*^−^ & *η*^+^, respectively) can be estimated (Miletić et al., 2020; Pedersen et al., 2017; Pedersen & Frank, 2020). Whereas negative prediction errors refer to outcomes that were worse than expected, positive prediction errors pertain to outcomes that were better than anticipated. In addition, the model also establishes how much evidence is needed before a decision is made (i.e., threshold separation, *a*), with larger (vs. smaller) values indicating greater response caution. Finally, the efficiency of non-decisional processes (e.g., stimulus encoding, response execution, *t*_0_) is also estimated.

In essence, RL models are applied to understand how feedback (i.e., reward or punishment) is utilised to update subjective expectations of associated outcomes and how, in turn, these revised beliefs guide behaviour (i.e., which response option will be selected in future trials; Fontanesi et al., 2019; Miletić et al., 2020; Pedersen & Frank, 2020). By applying the delta learning rule, the model describes the updating of the expected *Q*-value for a chosen option (e.g., positively reinforced symbol A) based on the scaled by learning rate (*η*) reward prediction error (i.e., the difference between observed and expected feedback) in the previous trial (Rescorla & Wagner, 1972; Watkins & Dayan, 1992, see Equation 1):

(1) $$\begin{array}{c} Q_{chosen-option}\left(t\right)\;=Q_{chosen-option}\left(t-1\right)\;\\ +\eta \left[Reward\;\left(t-1\right)\;-Q_{chosen-option}\left(t-1\right)\right] \end{array}$$

Learning can occur from two sources of prediction error (i.e., positive and negative). Whereas learning from positive prediction errors (*η*^+^) happens when the reward is better than expected, negative prediction errors (*η*^−^) describe updating when the outcome is worse than expected (Frank et al., 2007; Schultz, 2016). In such instances, based on the prediction error that occurred in a given trial, the *Q*-value is updated using the extended delta rule formulation (Frank et al., 2007; see Equation 2):

(2) $$\begin{array}{c} Q_{chosen-option}\left(t\right)\;=Q_{chosen-option}\left(t-1\right)\;+\eta^{+}\left(Reward\;\left(t-1\right)\;-Q_{chosen-option}\left(t-1\right)\right)\\ +\eta^{-}\left[Reward\left(t-1\right)\;-Q_{chosen-option}\left(t-1\right)\right] \end{array}$$

Two significant modifications characterise the RL-DDM compared with standard RL models. First, the typical choice rule for RL (i.e., softmax) is replaced by the Wiener diffusion process of the DDM (see Miletić et al., 2020; Pedersen et al., 2017; Pedersen & Frank, 2020). This is a crucial feature of the RL-DDM, as it affords the possibility to model choice and RT data simultaneously. Specifically, in the RL-DDM, the likelihood of each chosen option in a given trial and its associated RT are estimated using the standard DDM probability density function, the Wiener first passage time (wfpt) distribution (Navarro & Fuss, 2009; Pedersen & Frank, 2020). This function provides the probability of choosing option *i* in a given trial *t* and its observed RT *rt_i,t_* (see Equation 3):

(3) $$rt_{i,t}\sim wfpt(a,\;t_{0},\;v_{t})$$

Second, the algorithm that captures the learning of subjective expectation values (*Q*) from stimuli and actions (i.e., value-based approach) is integrated into the process of evidence accumulation (i.e., drift rate). Specifically, the RL-DDM formulates the drift rate (*v*) based on the difference between the expected value of positively (*Q*_positively-reinforced_) and negatively (*Q_negatively-reinforced_*) reinforced choices. To accommodate the way this knowledge is used, the RL-DDM allows an additional free scaling parameter to be estimated (i.e., drift rate scaling, *v*_scaling_). This scaling parameter is similar to the inverse temperature in the softmax choice rule and reflects the level of exploration/exploitation during learning (Pedersen & Frank, 2020), such that larger values reflect stronger exploitation of the option with the highest expected value. In the context of the current PST in which participants were presented with two stimuli on each trial and learned which one was most likely to be correct, exploration refers to the tendency to sample and consider both options, rather than consistently selecting the option that was previously associated with positive feedback (i.e., exploitation). See Equation 4:

(4) $$\begin{array}{c} v\left(t\right)\;=\;\left[Q_{positively-reinforced}\left(t\right)\;-Q_{negatively-reinforced}\left(t\right)\right]\;\\ {}*v_{scaling} \end{array}$$

As with other sequential-sampling models, the RL-DDM assumes that evidence is gathered for each choice option (e.g., symbol A vs. symbol B) until a critical evidential threshold is reached, at which point a response is made. This response threshold is captured by the boundary separation (*a*) parameter, which reflects speed–accuracy trade-offs during decision-making. For example, if a conservative (vs. liberal) decision-making style (i.e., higher evidential requirements) is adopted, this would yield slower but more accurate responses. At the start of the PST, participants make initial guesses as the stimuli have not yet been reinforced, thus the difference in expected values between symbol pairings is extremely low (i.e., slow evidence accumulation due to high uncertainty). As participants start to receive feedback, via application of the delta learning rule (Rescorla & Wagner, 1972), the subjective *Q*-learning values of positively/negatively reinforced stimuli increase/decrease. The speed at which participants update the expected values is described by the learning rate (*η*) parameter. On a trial-by-trial basis, this knowledge (i.e., learning which symbol is correct, *Q*-value) is integrated into the drift rate such that over time the difference in expected values between reinforced options (ACE vs. BDF symbol pairings) increases (Pedersen & Frank, 2020). The larger the difference between positively and negatively reinforced options, the easier (i.e., faster and more accurate) choice selection becomes (i.e., fast information sampling).

## Results

### Behavioural analysis

Based on the summary data, all participants fully completed the mindfulness or control (i.e., 4–6 Tangram puzzles per 5 min) exercises. For the PST, the outliers, which were omitted from the analyses, were responses faster than 200 ms or slower than 4,000 ms (Frank et al., 2007) and comprised approximately 1.5% of the data. The mean latency of responses and learning performance were submitted to JASP (JASP Team, 2023) for an independent-samples (Meditation: mindfulness or control) *t*-test (two-tailed). No significant differences emerged on either measure, that is, decision time: *M_mindfulness_* = 1,255 ms, *SD_mindfulness_* = 771 ms versus *M_control_* = 1,062 ms, *SD_control_* = 332 ms, *t(*53) = −1.18, *p* = .243; learning performance: *M_mindfulness_* = 73%, *SD_mindfulness_* = 7% versus *M_control_* = 74%, *SD_control_* = 8%, *t*(53) = 0.25, *p* = .804.

**Figure 2.**
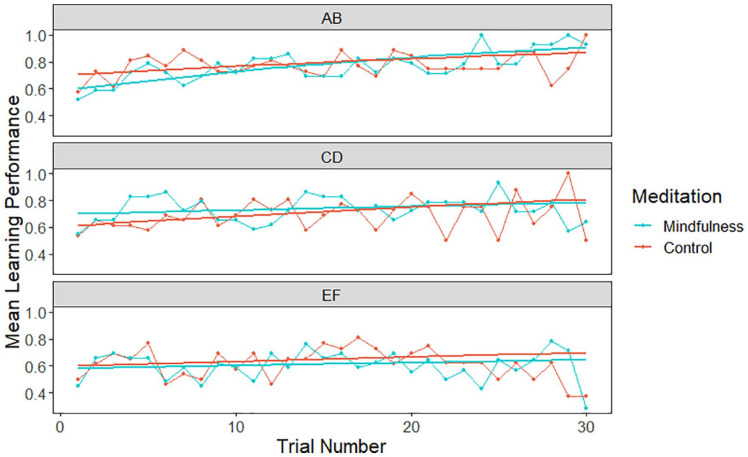
Based on ceiling performance during the PST, learning curves over the first 30 trials as a function of Meditation and Stimulus Pair were calculated. The raw data points represent the observed learning outcomes, and the modelled curves reflect the results of the generalised estimating equations (GEE) analysis. The GEE analysis examines differences in learning trajectories across trials and experimental conditions.

To examine the data more closely, using generalised estimating equations (GEE), a trial-by-trial analysis was conducted on the first 30 responses in the PST to explore the effects of Trial, Meditation (i.e., mindfulness vs. control), and Stimulus Pair (i.e., AB, CD, EF) on learning performance (see Figure 2). Analyses were conducted using the R package “geepack” (Højsgaard et al., 2006). A main effect of Trial, Wald χ^2^(1) = 4.49, *p* = .034, and a significant Meditation × Stimulus Pair “CD” interaction, Wald χ^2^(1) = 6.11, *p* = .013, were observed. Follow-up analyses revealed different dynamics in the mindfulness (vs. control) condition. Specifically, the interaction with the CD pair was significant, indicating that the effect of mindfulness on learning performance was more pronounced in this stimulus pair compared with the reference AB pair, Wald χ^2^(1) = 6.11, *p* = .013. For the EF stimulus pair, Wald χ^2^(1) = 0.92, *p* = .338, and the interaction with Trial for both the CD, Wald χ^2^(1) = 2.64, *p* = .104, and EF pair, Wald χ^2^(1) = 0.85, *p* = .356, no significant effects were observed. These results indicate that the influence of brief mindfulness meditation on learning performance varied across the stimulus pairs, with a specific effect observed for the CD pairing relative to the reference pair (AB).

### Modelling analysis

To identify the processes underpinning probabilistic learning and decision-making, data were fitted to a cognitive model (i.e., RL-DDM; Pedersen et al., 2017; Pedersen & Frank, 2020). To estimate model parameters, an extension of the Bayesian hierarchical drift diffusion toolbox was adopted (Wiecki et al., 2013). Models were response-coded, such that the upper threshold corresponded to positively reinforced stimuli (i.e., symbols corresponding to the letters A, C, and E) and the lower threshold to negatively reinforced items (i.e., symbols corresponding to the letters B, D, and F; Pedersen & Frank, 2020). As calculating the likelihoods of the posterior parameter values using Bayes’ theorem would be intractable (Wiecki et al., 2013), the posterior distributions were modelled using a Markov chain Monte Carlo (MCMC) with 10,000 samples (including 1,000 burn) using the Python package PyMC (see Patil et al., 2010). The MCMC approach samples potential parameter values based on the parameter prior distributions and assesses the likelihood of each sample, such that each new iteration is compared with the previous one and sampled more frequently if it provides a better estimation for the parameter values (Geyer, 2011). Across thousands of iterations, the resulting parameter posterior distributions will converge around the true parameter values. Outliers (5% of the trials) were removed by the HDDM software (Ratcliff & Tuerlinckx, 2002; Wiecki et al., 2013). Two RL-DDM models were estimated for comparison (i.e., single vs. dual learning rate model). In the first model, only a single learning rate (*η*) was allowed to vary across the experimental conditions (i.e., mindfulness vs. control). This model examined whether there were differences in the speed of learning without considering the different types of prediction error. In contrast, in the second model, learning rates for negative and positive prediction errors (*η−* and *η*^+^, respectively) were estimated separately and were allowed to vary as a function of experimental condition. As such, this model considered whether learning in each of the conditions accelerated following positive or negative prediction errors. In both models, drift rate scaling (*v*_scaling_) and boundary separation (*a*) varied as a function of experimental condition.

**Figure 3.**
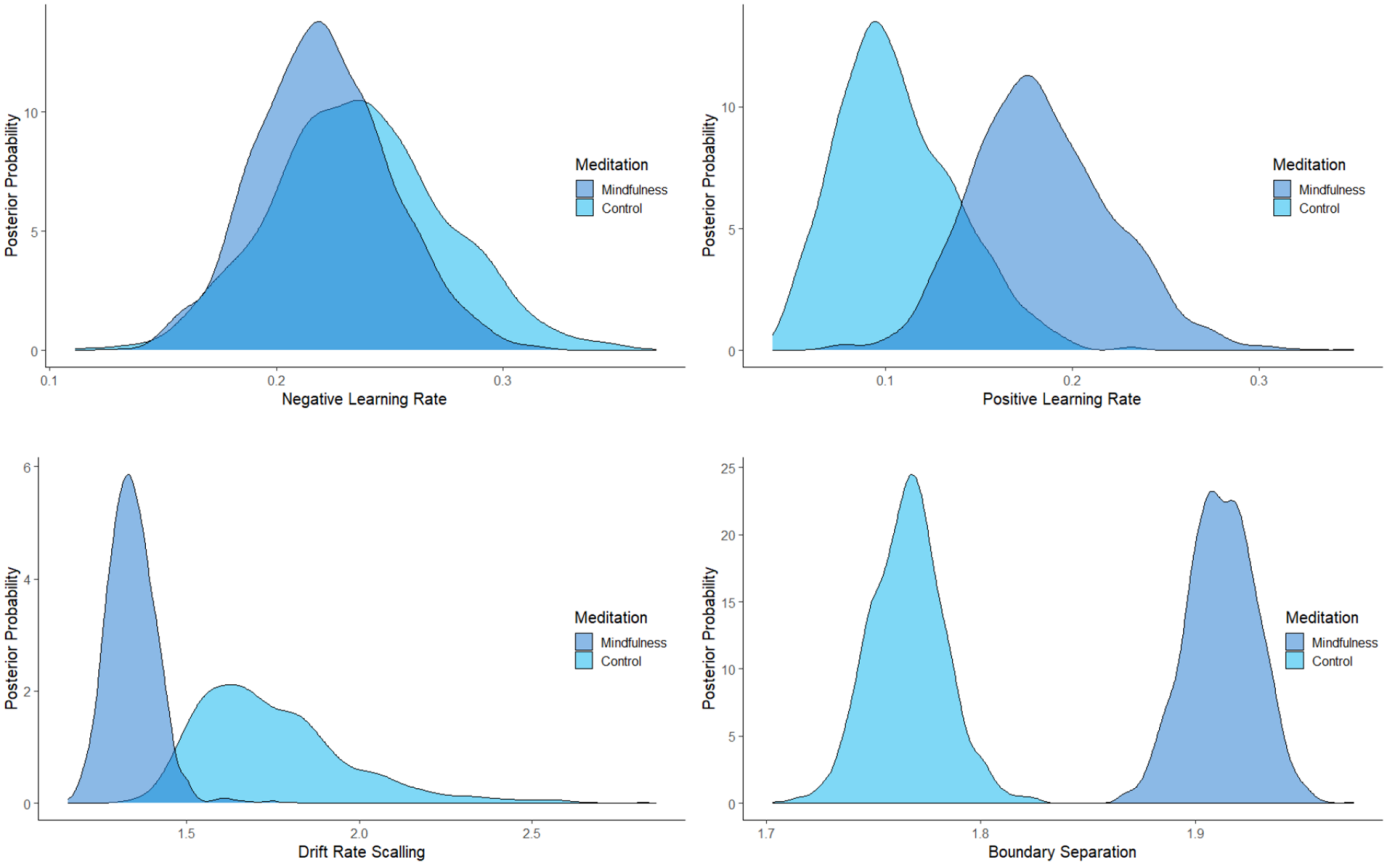
Posterior parameter distributions as a function of meditation for negative (*η−*) and positive (*η*^+^) learning rates, drift rate scaling (*v*_scaling_), and boundary separation (*a*).

Model comparison was performed using the deviance information criterion (DIC) as this approach is routinely adopted when comparing hierarchical Bayesian models (Spiegelhalter et al., 1998, 2002). Lower DIC values favour models with the highest likelihood and least number of parameters. This revealed better fit for the dual (DIC:16309) compared with the single (DIC: 16318) learning rate model. Examination of the posterior distributions (see Figure 3) indicated differences in learning rates for positive prediction errors (*η*^+^), drift rate scaling (*v*_scaling_), and threshold separation (*a*). Specifically, comparisons yielded strong evidence that learning rates were faster in the mindfulness condition for positive, *p*_Bayes_(mindfulness > control) = .061, BF_10_ = 15, but not for negative, *p*_Bayes_(mindfulness > control) = .377, BF_10_ = 2, prediction errors. There was also very strong evidence that drift rate scaling (*v*_scaling_) was smaller in the mindfulness condition, *p*_Bayes_(mindfulness < control) = .013, BF_10_ = 76. Finally, for boundary separation (*a*), there was extremely strong evidence that additional decisional evidence was required in the mindfulness condition, *p*_Bayes_(mindfulness > control) < .001, BF_10_ > 1,000, indicating that a brief period of meditation increased response caution.^4^

## Discussion

The current results demonstrated both the benefit of mindfulness-based meditation on probabilistic learning and the cognitive pathways through which this practice influenced decisional processing. Compared with their counterparts who performed a puzzle task (i.e., no meditation), participants who experienced a brief period of mindfulness-based meditation exhibited accelerated learning rates following positive (vs. negative) prediction errors. Interestingly, mindfulness-based meditation boosted performance for the medium probability stimulus pairing (i.e., CD), an effect that emerged during the early stages of the PST (see Figure 2). In terms of the operations underpinning decision-making, meditation increased response caution (i.e., additional evidence was required prior to response selection) and the tendency to explore (vs. exploit) choice selections (Frank et al., 2004, 2007). Collectively, these findings inform understanding of how brief mindfulness-based meditation influences instrumental learning.

Interestingly, both meditation and learning have been shown to modulate activity in the striatum, a core component of the dopaminergic system in the brain (Hagerty et al., 2013; Kjaer et al., 2002; McClure et al., 2003; Pagnoni et al., 2002). During learning, phasic bursts of dopamine act as a signal to orient people to novel or unexpected stimuli based on the discrepancy between the anticipated and actual outcome of an action (i.e., reward prediction error). Specifically, whereas dopamine levels spike following positive feedback, they correspondingly dip when feedback is negative (Schultz, 2016). In the current study, brief mindfulness-based meditation accelerated learning following positive (but not negative) prediction errors. That is, learning was enhanced when choice-related feedback was better than expected (i.e., choice selections were surprisingly correct). What this indicates is that mindfulness meditation increased participants’ responsivity to positive reward prediction errors, thereby accelerating the rate of learning during the PST.

On close inspection, the relationship between mindfulness and reward-based learning is complex, with processing influenced both by the specific requirements of the task at hand, situational constraints, and people’s levels of meditative expertise (e.g., Kirk & Montague, 2015; Kirk et al., 2019; Knytl & Opitz, 2019). Indeed, one intriguing possibility is that, at different dosages, mindfulness meditation may have the capacity either to enhance or dampen the signal value of reward prediction errors. Using a passive conditioning task, Kirk and Montague (2015) demonstrated attenuated reward prediction signals in the striatum among long-term meditators compared with matched controls. In follow-up research, this time testing participants undergoing an 8-week programme of mindfulness-based training, Kirk et al. (2019) similarly observed reduced responsivity to reward prediction errors during a conditioning task. As extensive meditative experience is associated with increased tonic dopamine levels (Kjaer et al., 2002), it is possible that the signal value of reward prediction errors is reduced for expert (vs. novice) mindfulness-based practitioners (Knytl & Opitz, 2019). In the current investigation, a brief period of mindfulness meditation accelerated the rate of learning following positive prediction errors. It remains to be seen, however, whether this effect would persist if participants experienced extended episodes of meditative training (i.e., multiple periods of brief mindfulness-based meditation may diminish the signal value of reward prediction errors). A useful task for future research will be to explore this issue.

In line with recent calls for computational approaches in psychiatry and meditation research (Hitchcock et al., 2023; Huys et al., 2016; van Vugt et al., 2019), the current investigation affirms the value of mathematical modelling. Unlike conventional statistical approaches, models of cognition (e.g., DDM) simultaneously analyse all the available data (i.e., full distributions of accurate and erroneous RTs as well as choices) to identify the latent psychological processes (e.g., response caution/bias, speed of information uptake) that underpin task performance (Evans & Wagenmakers, 2020; Miletić et al., 2020; Ratcliff et al., 2016). As such, this approach yields important insights into how mindfulness impacts cognition across different task contexts (e.g., meditation procedures, experimental paradigms). For instance, and corroborated by the current findings, an emerging observation is that, regardless of the task under consideration (e.g., attentional cueing, working memory, categorisation), mindfulness alters response caution (i.e., boundary separation, *a*; Golubickis et al., 2023; van Vugt & Jha, 2011; van Vugt & van den Hurk, 2017). Specifically, meditators change their decision-making style by requiring additional information to be accumulated before a judgement is made (i.e., conservative responding). Operating in this way, increased response caution may serve as one of the critical pathways through which the debiasing and de-centring effects of mindfulness-based mediation are realised (Golubickis et al., 2016, 2023; Hussain, 2015; Wells, 2005).

Importantly, the application of a computational model that combined two, characteristically independent, cognitive domains (i.e., instrumental learning and decision-making) allowed the decisional contaminants (e.g., speed–accuracy trade-off) that bias task performance to be controlled (Fontanesi et al., 2019; Miletić et al., 2020; Ratcliff et al., 2016). In so doing, the RL-DDM analysis explicated the cognitive pathways through which brief mindfulness-based mediation influenced instrumental learning. Moving forward, a similar approach could be applied to investigate other domains in which meditation impacts information processing. Take, for example, the common finding that mindfulness enhances attention (e.g., Jha et al., 2007; Semple et al., 2010; Valentine & Sweet, 1999). Paradigms that assess components of attention (e.g., selective attention, sustained vigilance, executive control) are also susceptible to a range of decisional biases (i.e., stimulus and response-driven processes; van Vugt et al., 2019). Crucially, however, such influences can be accounted for by implementing extensions of sequential sampling models that enable the specific attentional processes of interest to be estimated (e.g., shrinking spotlight diffusion model; Golubickis & Macrae, 2021; White & Curl, 2018; White et al., 2011).

The current study adopted a meditative practice that entails careful attention to one’s breathing and the cultivation of a non-judgmental awareness of thoughts and sensations (Chiesa, 2010; Hart, 1987). Notable psychological fingerprints of this practice include heightened meta-cognitive awareness, enhanced emotional regulation, and increased present-moment focus (Kok & Singer, 2017; Kropp & Sedlmeier, 2019). Our results extend these findings. Specifically, here we observed faster learning rates from positive prediction errors following brief meditation, suggesting heightened sensitivity to rewarding feedback during instrumental learning. In addition, we found that exploration was favoured by participants in the mindfulness condition, indicating their enhanced willingness to explore novel choice selections. Replicating prior research, participants also exhibited increased caution after mindfulness practice (Golubickis et al., 2023; van Vugt & Jha, 2011; van Vugt & van den Hurk, 2017). One possibility is that, via enhanced meta-cognitive awareness, diminished impulsivity increases the evidential requirements of response selection during decision-making.

Notwithstanding the reported findings, the current investigation is not without its limitations. Although revealing the effects of mindfulness on probabilistic learning, only a single meditative practice was adopted. Crucially, however, other mindfulness techniques, such as body scan and loving-kindness meditation have distinct psychological signatures, and thus may influence instrumental learning in different ways (Kok & Singer, 2017; Kropp & Sedlmeier, 2019). For instance, the body scan technique, which has been associated with increased self-compassion and enhanced emotional regulation (Kropp & Sedlmeier, 2019), may impact learning by influencing sensitivity to undesirable feedback (i.e., negative reinforcement). Relatedly, loving-kindness meditation, a practice allied with increased positivity and empathy (Kok & Singer, 2017) may influence learning through elevated responsivity to social rewards. Finally, also of interest is the issue of whether various mindfulness-based practices (e.g., guided vs. non-guided) exert comparable effects on response caution (i.e., evidential requirements of response selection). As different techniques wield sometimes divergent psychological effects, future research should explore how a range of meditative practices and dosages impact performance, potentially uncovering novel insights into the pathways through which mindfulness shapes decision-making and learning.

To establish the generalisability of the current findings, consideration should also be given to the effects of mindfulness-based meditation on other learning tasks. One such example is the probabilistic reward task in which participants learn responses that are associated with varying intermittent reinforcement schedules of monetary payoffs (Pizzagalli et al., 2005). Given alterations of the dopaminergic system following meditation (Hagerty et al., 2013; Kjaer et al., 2002; McClure et al., 2003; Pagnoni et al., 2002), it is possible that mindfulness may increase (or decrease) people’s sensitivity to reward incentives in such a task, thereby influencing the rate at which learning takes place. In addition, closer attention should be given to the characteristics of learning using paradigms which, unlike the PST, have been designed explicitly to assay the explore–exploit dilemma. For example, two/four-armed bandit tasks could be employed (Daw et al., 2006; Gershman, 2018). Finally, several psychiatric and neurological disorders, such as Parkinson’s disease, attention-deficit/hyperactivity disorder (ADHD), substance addiction, and schizophrenia, are associated with difficulties in RL (Frank et al., 2004; Huys et al., 2016; Maia & Frank, 2011). Future investigations should therefore explore whether mindfulness-based meditation can be used as an effective intervention in such cases.

In summary, probing the effects of mindfulness on RL, here we demonstrated the benefits of a brief period of mindfulness-based meditation on performance. Compared with their colleagues in the control condition, participants who underwent 5 min of mindfulness-based meditation displayed an accelerated rate of learning following positive reward prediction errors. In addition, learning was associated with greater response caution and a tendency to explore rather than exploit choice selections during decision-making (Frank et al., 2004, 2007). These findings confirm the value of computational approaches in elucidating the mechanisms through which mindfulness-based meditation influences instrumental learning.

## Notes

1. Instrumental learning is a form of associative learning in which an individual’s behaviour is modified based on the consequences (i.e., outcomes) of prior actions (i.e., choices).

2. It was not possible to probe these individual differences in the current investigation. Accordingly, how exactly prior meditative experiences (and theoretically motivated individual differences) influence instrumental learning is an issue that merits future consideration.

3. The data for the experiment are publicly accessible at: https://osf.io/xud5c/. The code for the RL-DDM analysis was taken from: ttps://hddm.readthedocs.io/en/latest/demo_RLHDDMtutorial.html.

4. Bayesian *p* values quantify the degree to which the difference in the posterior distribution is consistent with the hypothesis. For example, a Bayesian *p* of .05 indicates that 95% of the posterior distribution supports the hypothesis that the parameter posteriors differ across the conditions. Bayes factors (i.e., BF_10_) were calculated following Marsman and Wagenmakers (2017) with values >10 indicating strong evidence for a comparison (Wagenmakers et al., 2011).
